# Brain regions concerned with the identification of deceptive soccer moves by higher-skilled and lower-skilled players

**DOI:** 10.3389/fnhum.2013.00851

**Published:** 2013-12-17

**Authors:** Michael J. Wright, Daniel T. Bishop, Robin C. Jackson, Bruce Abernethy

**Affiliations:** ^1^Department of Psychology, Centre for Cognition and Neuroimaging, Brunel UniversityUxbridge, Middlesex, UK; ^2^School of Sport Sciences and Education, Centre for Sports Medicine and Human Performance, Brunel UniversityUxbridge, Middlesex, UK; ^3^Faculty of Health Sciences, University of QueenslandBrisbane, QLD, Australia; ^4^Institute of Human Performance, University of Hong KongPokfulam, Hong Kong, China

**Keywords:** fMRI, action observation, deception, expertise, soccer, football, mirror neuron system, sport

## Abstract

Expert soccer players are able to utilize their opponents' early body kinematics to predict the direction in which the opponent will move. We have previously demonstrated enhanced fMRI activation in experts in the motor components of an action observation network (AON) during sports anticipation tasks. Soccer players often need to prevent opponents from successfully predicting their line of attack, and consequently may try to deceive them; for example, by performing a step-over. We examined how AON activations and expertise effects are modified by the presence of deception. Three groups of participants; higher-skilled males, lower-skilled males, and lower-skilled females, viewed video clips in point-light format, from a defender's perspective, of a player approaching and turning with the ball. The observer's task in the scanner was to determine whether the move was normal or deceptive (involving a step-over), while whole-brain functional images were acquired. In a second counterbalanced block with identical stimuli the task was to predict the direction of the ball. Activations of AON for identification of deception overlapped with activations from the direction identification task. Higher-skilled players showed significantly greater activation than lower-skilled players in a subset of AON areas; and lower-skilled males in turn showed greater activation than lower-skilled females, but females showed more activation in visual cortex. Activation was greater for deception identification than for direction identification in dorsolateral prefrontal cortex, medial frontal cortex, anterior insula, cingulate gyrus, and premotor cortex. Conversely, greater activation for direction than deception identification was found in anterior cingulate cortex and caudate nucleus. Results are consistent with the view that explicit identification of deceptive moves entails cognitive effort and also activates limbic structures associated with social cognition and affective responses.

## Introduction

Expert players in interceptive sports such as soccer react under great time pressure and therefore, need to predict the actions of their opponents and the direction of play (Reilly et al., 2000; Abernethy et al., 2001; Savelsbergh et al., 2005; Williams et al., 2011). Studies have shown that superior analysis of body kinematics underpins much anticipation skill in sport. In the temporal occlusion paradigm, action is cut off at various time intervals relative to a crucial event (such as point of direction change in soccer), and the observer judges the direction of the shot. Results consistently show that experts are able to detect the predictive information with greater accuracy and earlier than novices (Abernethy and Russell, 1984, 1987; Abernethy et al., 2008). The nature of the predictive information has also been identified using techniques such as spatial occlusion, in which different parts of the opponent's body are systematically masked (Muller et al., 2006; Jackson and Mogan, 2007). The reductive approach to identifying the minimum visual information sufficient to support expert anticipation has been taken further with the use of point-light video stimuli. Comparisons of performance based on ball trajectory alone and studies using point-light stimuli indicate the pre-eminence of body kinematics as a cue to future action (Abernethy et al., 2001, 2008; Huys et al., 2008).

A general conclusion from this research is that experts are better than novices at detecting predictive cues in opponents' body kinematics, and this gives them an advantage in speed and accuracy. Precisely for this reason, skilled players also need to develop strategies to reduce the predictability of their own actions. The effectiveness with which deceptive moves can thwart anticipation has been established in soccer (Dicks et al., 2011; Smeeton and Williams, 2012; Bishop et al., 2013); rugby football (Jackson et al., 2006; Brault et al., 2012; Mori and Shimada, 2013); basketball (Sebanz and Shiffrar, 2009; Kunde et al., 2011); handball (Cañal-Bruland and Schmidt, 2009; Cañal-Bruland et al., 2010), and tennis (Rowe et al., 2009). In these studies it was found that experts are more accurate than novices in predicting the outcome of deceptive moves, and that the expert-novice difference tends to be greater for deceptive than for normal moves.

Neuroimaging studies have provided some insights into the neural structures that mediate anticipation skills. A substantial literature has developed around functional imaging studies of cortical networks that mediate the perception of, and the production of responses to, others' actions. Molenberghs et al. (2012) conducted a meta-analysis of 125 fMRI studies of the human “mirror neuron system” (MNS) and identified a core network of brain areas including inferior frontal gyrus, dorsal and ventral premotor cortex, and inferior and superior parietal lobule, that were activated in studies involving the observation and/or production of actions. Most fMRI experiments on the observation of actions do not include direct evidence for the presence of mirror neurons, so we refer in the present paper to an action observation network (AON: Grafton, 2009) rather than MNS. The AON does nevertheless include the structures identified by Molenberghs et al. (2012) as core elements of the MNS.

Research has demonstrated the importance of the AON in sport, including structures traditionally interpreted as having motor functions. Wright and Jackson (2007) measured cortical fMRI activation in predicting the direction of a tennis serve from temporally-occluded video clips. Relative to a passive, action-observation control condition, action prediction activated the anterior components of the AON, particularly the dorsal and ventral premotor cortex. Aglioti et al. (2008) found that observation of basketball shots increased the strength of motor-evoked potentials elicited by transcranial magnetic stimulation, and that experts showed a time-specific motor activation for missed shots, indicating a close and specific interaction between perceptual and motor systems that is dependent on experiential learning. Wright et al. (2010) found stronger activation for expert badminton players while predicting the direction of badminton shots in components of the AON, specifically, medial frontal cortex, inferior frontal gyrus, anterior insula, and superior parietal lobule. Wright et al. (2011) showed that low-resolution point-light badminton video effectively supported judgments of the direction of a shot, and elicited a corresponding full pattern of fMRI activations in these areas including expertise effects, thus, indicating the sufficiency of body kinematics as input to the AON. Bishop et al. (2013) studied neural correlates of direction prediction in soccer, with temporally-occluded video stimuli that included deceptive moves, and with randomized presentation that maximized uncertainty. High-skilled observers showed stronger responses than intermediates and novices not only in cortical AON structures but also in subcortical structures, including cerebellum, lentiform nucleus, and thalamus, that have been implicated in response selection (Yarrow et al., 2009).

Correct direction prediction in a situation where an opponent can use deceptive moves, for example when an oncoming rugby player executes a side-step, may involve attending to “honest” movement cues and ignoring “deceptive” movement signals (Brault et al., 2012). This is a complex skill that entails more than simply being able to recognize a normal or a deceptive move: the correct implication of that move in terms of outcome (future direction of play) must also be perceived or comprehended. This is perhaps the reason that highly-skilled players often take longer to react than novices in the presence of deception, and achieve greater accuracy as a result (Brault et al., 2012; Mori and Shimada, 2013). In some studies, experts are found to be significantly disadvantaged by deception, notwithstanding they may be less disadvantaged than novices (Brault et al., 2012; Bishop et al., 2013; Mori and Shimada, 2013). Possible reasons for this include an increased cognitive load, perceptual uncertainty, or misdirection of attention in the presence of deception.

The purpose of the present study was to analyze the neural and behavioral responses of lower-skilled and higher-skilled players to the task of identifying soccer moves as normal or deceptive, and by comparison, measuring the neural and behavioral response to identifying future direction of play in an identical (normal plus deceptive) stimulus set. Most studies of deceptive moves in sport have used identification of future direction of play as a measure (Jackson et al., 2006; Rowe et al., 2009; Dicks et al., 2011; Kunde et al., 2011; Brault et al., 2012; Smeeton and Williams, 2012; Bishop et al., 2013; Mori and Shimada, 2013). A smaller number have measured deception identification (Cañal-Bruland and Schmidt, 2009; Sebanz and Shiffrar, 2009; Cañal-Bruland et al., 2010). These tasks are not equivalent. Firstly, as Cañal-Bruland and Williams (2010) found, the kinematic information used when predicting the direction of a shot differs from that used when discriminating between two different movement patterns. Secondly, the consequences of the judgment are different. Direction identification requires a directional or spatial judgment with implications for the direction of an interceptive movement. Equally, deception identification implies a more analytical judgment of an observed action as having some goal or intent, but without specifying direction. It was therefore, hypothesized that both behavioral performance and cortical patterns of activation for direction identification and deception identification may differ, and that there would be differences in the activation of task-related regions, as identified by fMRI, in lower-skilled and higher-skilled players. In view of the research reviewed above showing the sufficiency of body kinematics in sport action prediction tasks, and in order to eliminate irrelevant stimulation by background stimuli, physical appearance and clothing of actors, we utilized point-light stimuli for the tasks.

## Materials and methods

### Experimental design and procedure

In a block-design, fMRI study, participants in the scanner viewed 2-s video sequences of an opposing soccer player dribbling the ball toward the viewer, and pressed a button to indicate which direction the player would turn; that is, the leftmost button for a turn to the observer's left, and the rightmost button for a turn to the observer's right. There was an interstimulus interval of 2-s during which a gray screen at mean luminance was present, and instructions were to respond as accurately as possible during the interstimulus interval. There were five video clips in each block. Exactly half of the sequences of each type were based on deceptive moves (step-over) and half on normal moves, both for direction prediction and for control conditions. The type of move (normal or deceptive) was randomized within blocks. In addition to fMRI data, button press responses were recorded and analyzed for accuracy.

A second session of the experiment utilized exactly the same stimulus material and block design but required a different action identification task: instead of predicting which direction the player would turn, the observer had to indicate by a button press whether a move was normal or deceptive. The order of sessions was counterbalanced across participants.

For both action identification tasks, we used a control block: a single static frame at the start of the point-light footballer's run was used, and it was slowly magnified (zoomed) over 2-s to match the apparent motion of the footballer toward the observer. However, as it was derived from a static frame, there was no biological motion: that is, there was no relative motion between the dots representing the movements of the footballer's limbs and trunk. We therefore, refer to this as a non-biological motion (NBM) control. The required response for this type of video was simply to press a middle button. Mean accuracy on this task was 99.9%. A further type of block required participants to respond to an altered dot in the point-light footballer video (98.5% correct): but further analyses of the responses to this condition are not within the scope of the present paper.

Before each block of 5 trials, a 5-s instruction screen appeared specifying the task for the subsequent block. Blocks were presented in a fixed pseudorandom sequence. Altogether there were eight repetitions of the three types of block: (1) soccer direction identification with 0 ms occlusion; (2) soccer direction identification with −160 ms occlusion; (3) NBM control. The total duration including instruction screens and blank intervals was 18 min.

The same control task and stimuli were used in the deception identification session as in the direction identification session. Thus, the only difference in the material for the two versions of the experiment was in the on-screen instructions. The three types of block were thus, (1) soccer deception identification with 0 ms occlusion; (2) soccer deception identification with −160 ms occlusion; (3) NBM control. Participants undertook both versions of the experiment, and the order was counterbalanced between experiments. The experiment as a whole comprised two 18-min sessions, plus an anatomical scan lasting 5 min.

After completing the pre-scan screening and informed consent procedures, participants were instructed in the nature of the task and shown examples of the stimuli. They were asked whether they were familiar with the step-over as a deceptive move, and if not, a brief verbal explanation was given.

### Participants

The participants were 17 higher-skilled male soccer players (mean age 22.6, *SD* 4.0, range 19–33 years), 17 lower-skilled male soccer players (mean age 22.1, *SD* 3.7, range 19–31 years). Additionally 17 females (mean age 20.1, *SD* 1.1, range 19–23 years) were included as a group with minimal soccer experience. Participants were recruited by advertising on University notice-boards and websites and by word of mouth and were offered £20 in expenses to recompense for their time and inconvenience. All participants gave their written informed consent as part of a protocol approved by the Brunel University Department of Psychology Ethics Committee. Procedures for fMRI were conducted according to the Rules of Operation of the Combined Universities Brain Imaging Centre. All participants completed a questionnaire giving brief demographic details and providing information on their soccer experience and expertise. Higher-skilled players were defined as those playing currently or within the last year in a league with regular fixtures and for a named club whose provenance could be checked on the internet. They were drawn from local leagues and University teams and did not include elite or professional players. Lower-skilled players were nonplayers or recreational players, but included some with previous experience (more than 1 year previous) of playing competitively for local sports clubs or school teams. All but one participant in the lower-skilled male group had played soccer in childhood. Table 1 compares the samples according to age and soccer experience. Higher- and lower-skilled males differed significantly on a Mann-Whitney *U* test in the highest level of competition achieved; *U* = 29, *p* < 0.0005, the number of hours per week in training; *U* = 50, *p* < 0.005; and the number of matches watched (live or on television or other media) per month, *U* = 105, *p* < 0.05. They did not differ significantly in age, in the age at which they started playing, or in the skill level of other sports played. The lower-skilled females differed significantly from the lower-skilled males in the number of years playing, *U* = 82, *p* < 0.005; competitive level, *U* = 116, *p* < 0.05; hours per week training, *U* = 111, *p* < 0.05 and matches watched per month, *U* = 70, *p* < 0.005. They did not differ significantly in the level of other sports played. From the point of view of the research hypotheses, the female lower-skilled group provides a baseline with a low level of soccer experience: the possible influence of gender will be addressed in the Discussion.

**Table 1:** **Comparison of the soccer experience of the participant groups**.

	**Years playing**	**Median competitive level**	**Hours training per week**	**Matches watched per month**	**Median level of main other sport played**
Higher-skilled males	*M* = 13.6	Local league	*M* = 4.5	*M* = 8.5	Recreational
	*SD* = 4.2		*SD* = 3.4	*SD* = 5.3	
Lower-skilled males	*M* = 8.9	None	*M* = 1.5	*M* = 5.1	Recreational
	*SD* = 7.6		*SD* = 2.5	*SD* = 4.5	
Lower-skilled females	*M* = 0.5	None	*M* = 0.1	*M* = 0.6	Recreational
	*SD* = 1.4		*SD* = 0.3	*SD* = 0.9	

### Stimuli

All experiments utilized 2-s point-light video clips of three junior international male soccer players dribbling the ball toward a video camera (NV GS400; Panasonic Corporation, Secaucus, NJ) placed at a distance of 11.5 m from the start of the player's run, in an indoor sports hall. The actors ran toward the camera, then at a predetermined point, moved obliquely to the left or right as they would in evading a defending player's interception. They performed a deceptive maneuver known as a step-over in 50% of runs immediately prior to a direction change. The color video was edited (Pinnacle Studio Pro v 11.0, Pinnacle Systems, CA) frame by frame to produce sparse binary (black/white) point-light representations consisting of 15 small disc markers on principal body joints and extremities. The ball was represented in each frame by a white disc. There was no representation of surface texture, depth, orientation, or color, either that of the player or that of the background. To generate different levels of temporal occlusion, the video was truncated at various time points relative to the passing of the floor marker (0 ms). Two occlusion levels were used (−160 and 0 ms).

### Acquisition and analysis of fMRI data

Functional and structural images were acquired on a MAGNETOM Trio 3T MRI scanner (Siemens Medical Solutions; Bracknell, UK) using Siemens' parallel imaging technology (iPat), which was deployed with a generalized auto calibrating partially parallel acquisitions (GRAPPA) acceleration factor of two, via a Siemens eight-channel array head coil. For each functional run, an ultra-fast echo planar gradient-echo imaging sequence sensitive to blood-oxygen-level dependent (BOLD) contrast was used to acquire 41 transverse slices (3 mm thickness) per TR (3000 ms, TE 31 ms, flip angle = 90°). For each version of the experiment, 360 volumes were acquired in a 192 × 192 mm field of view with a matrix size of 64 × 64 mm, giving an in-plane spatial resolution of 3 mm (generating 3 mm^3^ voxels). Anatomical data were collected in the same orientation and plane as the functional data to enable localization, using an MP-RAGE T1-weighted sequence, in which 176 one-mm slices alternated with a 0.5 mm gap. The structural sequence incorporated 1830 ms TR, 4.43 ms TE, FoV 256 mm and a GRAPPA acceleration factor of two.

#### Data acquisition and preprocessing

fMRI data were analyzed using the batch processing utilities of SPM8 (http://www.fil.ion.ucl.ac.uk/spm/). Functional images for both sessions were spatially realigned by initially aligning the first images of each session, and then aligning the images within each session to the first image, to moderate the effects of participants' head motion. Images were normalized using the SPM8 EPI template to account for anatomical variability, and to facilitate reporting of activation sites in the Montreal Neurological Institute (MNI) standard space. Finally, data were smoothed using a Gaussian kernel of 6 mm full-width half-maximum (FWHM) to increase the signal-to-noise ratio according to the matched filter theorem. The selected design matrix convolved the experimental design with a hemodynamic response function to model the hemodynamic lag behind the neuronal response. This model was estimated using proportional scaling over the session to remove global effects, and with a high pass filter of 128 s.

#### Statistical analysis

Individual level whole-brain fMRI *t*-contrasts were computed between experimental and control conditions as follows: (1) 0 ms occlusion vs. NBM control, (2) −160 ms occlusion vs. NBM control. This analysis was repeated for both direction identification and deception identification. Second-level, group data were analyzed using the SPM8 full factorial ANOVAs procedure. Two ANOVAs were conducted: the first, within-group ANOVA was based on the first level contrast between the experimental tasks and the NBM control and it was carried out twice, once for each group. The purpose of this analysis was to identify the within-group patterns of activation for the two tasks, deception and direction identification. Between-group differences were analyzed in the main (3 × 2 × 2) mixed ANOVA; the three factors were *expertise* (higher-skilled males, lower-skilled males, lower-skilled females), *task* (deception identification, direction identification), and *occlusion* (0, −160 ms). The input data to the ANOVA model were the first-level *t*-contrasts for 0 ms and for −160 ms occlusion vs. NBM control, for both the deception and direction identification tasks. Family-wise error (FWE) correction was used for all whole brain data. Identification of the location of peaks and clusters and assignment of Brodmann area (BA) labels was carried out in MNI space using WFU-Pickatlas (Maldjian et al., 2003). Accuracy of behavioral responses in the scanner was also analyzed statistically: details are given below.

## Results

### Behavioral results

#### Identification accuracy

The percentage of correct responses was measured for both direction identification and deception identification in the scanner, and a mixed ANOVA was conducted with identification task (deception, direction), trial type (normal, deceptive), and occlusion (0, −160 ms) as within-participant variables and group (higher-skilled male, lower-skilled male, lower-skilled female) as a between-participant variable. There was a significant main effect of trial type, *F*_(1, 48)_ = 63.5, *p* < 0.0005, η^2^_*p*_ = 0.59; with the mean accuracy higher for normal, *M* = 75.8% than for deceptive trials, *M* = 53.6%. There was also a significant main effect of occlusion, *F*_(1, 48)_ = 64.5, *p* < 0.0005, η^2^_*p*_ = 0.59, with higher accuracy for late occlusion (*M* = 72.4%) than for early occlusion (*M* = 57.6%). There was also a significant main effect of group, *F*_(2, 48)_ = 10.4, *p* < 0.005, η^2^_*p*_ = 0.32. Tukey's HSD showed that higher-skilled males (*M* = 72.7) differed significantly in overall accuracy from lower-skilled males (*M* = 64.5%, *p* < 0.05) and lower-skilled females (*M* = 57.9%, *p* < 0.001). Lower-skilled males and females did not differ significantly from one another. It was also expected that higher-skilled participants would be relatively superior in their response to deceptive stimuli, and this was confirmed; the interaction of expertise and trial type was significant, *F*_(2, 48)_ = 3.9, *p* = < 0.05, η^2^_*p*_ = 0.15. These results are broadly consistent with previous work on expertise and anticipation skill. Additionally, normal and deceptive stimuli were differentially affected by occlusion, thus, for trial type x occlusion, *F*_(1, 48)_ = 25.7, *p* < 0.0005, η^2^_*p*_ = 0.39.

**Figure 1:**
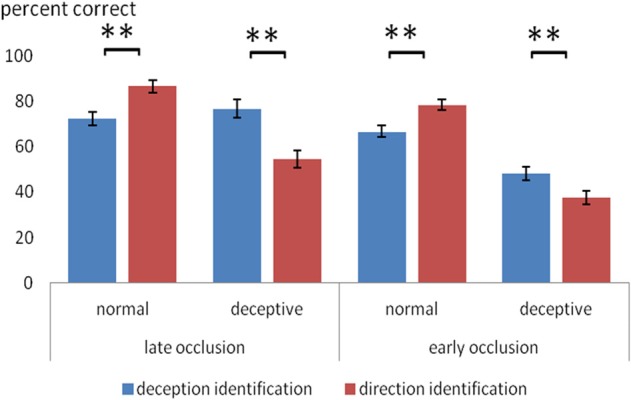
**Mean percentage accuracy on normal and deceptive trials in scanner sessions where the task was to identify of the type of move (normal or deceptive) and in sessions where the task was to identify the direction of play (left or right)**. Error bars are ±1 s.e.m. Difference between deception identification and direction identification (bracketed bars) is significant at ^**^p < 0.005.

A novel aspect of the design was the comparison of two different identification tasks. Overall, the two tasks had a similar level of difficulty: for deception identification (*M* = 65.7%) and for direction identification (*M* = 64.3%) and the overall difference in accuracy was not significant. However, there was a significant two-way interaction between identification task (deception, direction) and trial type (normal, deceptive), *F*_(1, 48)_ = 28.7, *p* < 0.0005, η^2^_*p*_ = 0.39. As shown in Figure 1, on normal moves, accuracy was significantly higher for direction identification, but on deceptive moves, accuracy was significantly higher for deception identification. There was also a significant three-way interaction between identification task, trial type and occlusion, *F*_(1, 48)_ = 13.2, *p* < 0.005, η^2^_*p*_ = 0.22, such that the task difference on deceptive trials was greater on late-occluded than early-occluded blocks. These interactions are of particular interest and to aid their interpretation, the mean scores are shown in Figure 1.

Figure 1 shows that for normal trial stimuli, mean accuracy of identification was relatively high (66–86%), both for early and late occlusion. For deceptive trial stimuli, the results were more complex. Planned comparisons (within-participants *t*-tests) were carried out for all comparisons of deception identification and direction identification, and the significant results are shown in Figure 1. Thus, for late occluded stimuli, participants performed with 76% accuracy in identifying deceptive stimuli on deceptive trials, but on judging direction with the same stimuli their accuracy (54%) was not significantly better than chance on a one-sample *t*-test.

#### Signal detection theory analysis

Because the percentage correct accuracy values may be affected by response bias, signal detection theory (SDT: Green and Swets, 1966) was applied. This method has been used previously to analyze the identification of normal vs. deceptive movements by Cañal-Bruland and Schmidt (2009). SDT calculates two variables, d-prime (d′: perceptual sensitivity), and beta (β: likelihood ratio or response bias). The d′ is a measure of the difference between the signal and noise distributions, calculated as d′= *z*(H) - *z*(F), where H is “hits” or correct identifications, and F is false positives, expressed in terms of their common standard deviation (*z*-units) (Macmillan and Creelman, 2005). Thus, for deception identification, H was taken to be the proportion of correct identifications of normal moves, and F was taken to be the proportion of deceptive moves incorrectly identified as normal. For direction identification, H was taken to be the proportion of correct identifications of direction on normal moves, and F was taken to be the proportion of incorrect identifications of direction on deceptive moves.

For deception identification, d′ was significantly greater for late-occluded stimuli (*M* = 1.46) than for early-occluded stimuli (*M* = 0.59). There was also a significant main effect of expertise. *Post-hoc* contrasts (Tukey) showed that the higher-skilled males were more sensitive to the difference between normal and deceptive moves than the lower-skilled males, and lower-skilled females.

**Figure 2:**
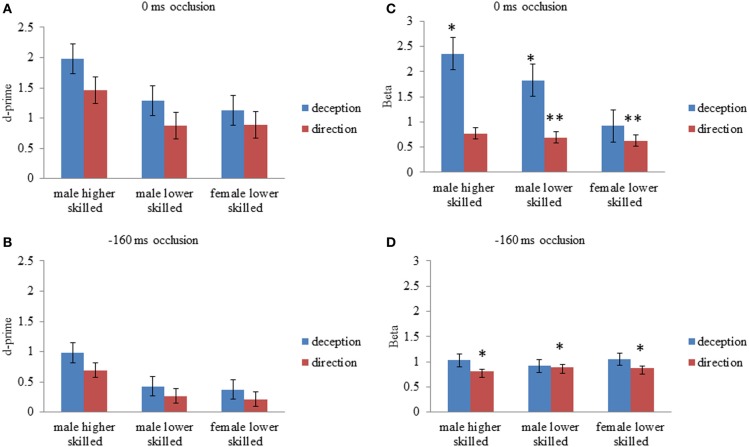
**Mean values for d-prime (d′); perceptual sensitivity, and Beta (β); likelihood ratio or response bias, for all experimental conditions**. **(A)** d′, 0 ms occlusion; **(B)** d′, −160 ms occlusion; **(C)** β, 0 ms occlusion **(D)** β, −160 ms occlusion. Error bars are ±1 s.e.m. Asterisks for Beta values represent mean values significantly different from 1 (one sample *t*-test, two-tailed *p* < 0.5^*^ < 0.005^**^). All d′ were significantly different from zero (one sample *t*-test, two-tailed).

For direction identification, d′ was significantly greater for stimuli occluded at 0 ms (*M* = 1.07) than for stimuli occluded at −160 ms (*M* = 0.39). There was also a significant main effect of expertise and *post-hoc* contrasts (Tukey) showed that the higher-skilled males were more sensitive to direction than the lower-skilled males or females, taking into account the false identifications of direction on deceptive moves. Although raw accuracy scores on normal moves were higher for direction identification (Figure 1), overall sensitivity to direction (d′) was lower than for deception identification, because of the incorrect responses on deceptive moves (Figure 2).

The criterion position *c* is the midpoint of the normalized hits and false positives, *c* = −1/2[*z*(H) + *z*(F)]. A more generally accepted measure of response bias is the likelihood ratio (β) which takes sensitivity (d′) into account, and is calculated as *e*^*cd*′^, where cd′ = −1/2 [*z*(H)^2^ − *z*(F)^2^] (Macmillan and Creelman, 2005; Cañal-Bruland and Schmidt, 2009). A neutral criterion is *c* = 0, or β = 1. For deception identification, if an observer were biased toward identifying moves as normal, then this would increase both the hits and the false positives, and give β < 1, that is, a liberal criterion. If the observer were biased toward identifying moves as deceptive, it would decrease both the hits and the false positives and give β > 1, that is, a conservative approach to identifying a normal shot.

For deception identification ANOVA showed a significant main effect of occlusion (Table 2), with larger β indicating a conservative criterion for late-occluded (*M* = 1.7) but not for early-occluded stimuli (*M* = 0.39). There was also a significant main effect of expertise. *Post-hoc* comparison showed that higher-skilled males set their criterion significantly further toward deception, relative to lower-skilled females. The interaction between expertise and occlusion was also significant with the expertise difference appearing on late-occluded stimuli (Figure 2).

**Table 2:** **Significant results from ANOVA conducted separately for deception identification and direction identification**.

	**Condition**	***F***	***df***	***p***	**η^2^_*p*_**	***Post-hoc* (Tukey)**
Deception identification d'	Occlusion	48.8	1,48	^***^	50	
	Expertise	4.9	2,48	^*^	0.17	HSM > LSM, *p* < 0.05
						HSM > LSF, *p* < 0.05
Deception identification β	Occlusion	11.7	1,48	^**^	0.20	
	Expertise	4.5	2,48	^*^	0.15	HSM > LSF, *p* < 0.05
	Occ. × Exp.	4.5	2,48	^*^	0.15	
Direction identification d'	Occlusion	36.3	1,48	^***^	0.43	
	Expertise	4.1	2,48	^*^	0.15	HSM > LSM, *p* < 0.05
						HSM > LSF, *p* < 0.05
Direction identification β	Occlusion	6.0	1,48	^*^	0.11	

Means and standard errors for every cell are shown in Figure 2. In each case, the independent variables were temporal occlusion (0 ms, −160 ms), and expertise (HSM, higher-skilled males; LSM, lower-skilled males; LSF, lower-skilled females). p < 0.05 ^*^ < 0.005 ^**^ < 0.0005^***^.

For direction identification the interpretation of β is a little more complex, as it represents a perceptual bias rather than a response bias. A value of β < 1 implies incorrect identification of direction on deceptive moves (designated false positives), without a corresponding increase in errors on normal moves (designated misses), that is, a tendency to analyze the direction of all moves as if they were normal. This would arise if deceptive cues that are incongruent with direction resemble normal cues that are congruent with direction. A value of β > 1, conversely, would represent a tendency to err on normal moves but not on deceptive moves; in effect, to treat appearances as deceptive. Thus, some of the effect of deception is revealed on the criterion measures. Figure 2 shows that all cell mean β values for direction identification were less than 1 and the planned comparisons (one-sample *t*-tests) were significant separately for all experimental conditions except for higher-skilled players on late-occluded stimuli: there was a general tendency to treat appearances as normal and to be fooled by deceptive moves. The main effect of expertise on β was not significant: there was no evidence that higher-skilled players adopted a different criterion from lower-skilled players. The only significant ANOVA result for β in direction identification (Table 2) was a main effect of occlusion, with β smaller for late-occluded (*M* = 0.70) than early occluded stimuli (*M* = 0.86).

It can be concluded that higher skilled males were significantly more sensitive to the cues that differentiate normal from deceptive soccer moves. However, they also showed a bias toward identifying moves as deceptive, and in this respect they resembled the skilled handball goalkeepers in the study of Cañal-Bruland and Schmidt (2009). Results also showed that higher-skilled males were significantly more sensitive overall than lower-skilled observers to directional cues. However, observers on average adopted a liberal criterion for direction identification (Figure 2), which is a significant tendency to treat deceptive moves like normal moves for the purposes of identifying direction, in other words, to be fooled by the deceptive stimuli.

### Whole-brain analysis of fMRI data

#### Within-groups analysis

**Figure 3:**
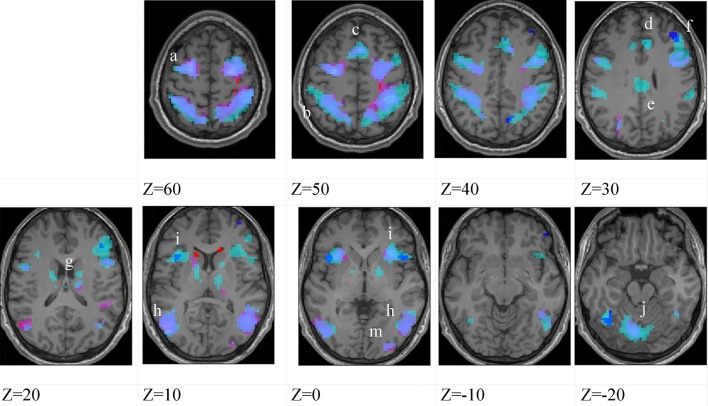
**Higher-skilled males**. Second-level fMRI activations (*p* < 0.005, FWE corrected, 25 voxels minimum cluster size) to deception identification (cyan) and direction identification (magenta) in point-light soccer video clips, relative to stimulus-matched non-biological motion (NBM) controls. Overlapping areas responding to both identification tasks appear purple. Activations above threshold (blobs) are displayed in co-registration with an individual normalized structural brain image and sampled in horizontal sections 10 mm apart from *z* = 60 to *z* = −20. In darker blue areas, activation to deception identification exceeds activation to direction identification; and in red areas, activation to direction identification exceeds activation to deception identification (at *p* < 0.001 uncorrected). Key: a: premotor, BA6; b: parietal, BA40; c: medial frontal, BA6; d: anterior cingulate, BA32; e: posterior cingulate, BA23; f: dorsolateral prefrontal, BA46; g: caudate nucleus; h: superior temporal gyrus, BA37; i: anterior insula/frontal operculum, BA13/45; j: cerebellum; k: superior parietal lobule, BA7.

**Figure 4:**
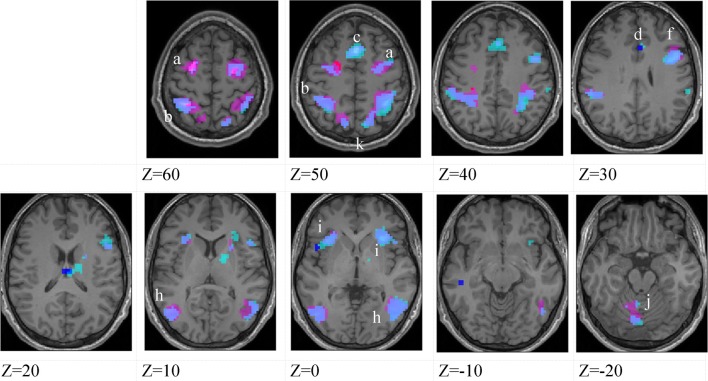
**Lower-skilled males**. Second-level fMRI activations (*p* < 0.005, FWE corrected, 25 voxels minimum cluster size) to deception identification (cyan) and direction identification (magenta) in point-light soccer video clips, relative to stimulus-matched non-biological motion (NBM) controls. Overlapping areas responding to both identification tasks appear purple. Activations above threshold (blobs) are displayed in co-registration with an individual normalized structural brain image and sampled in horizontal sections 10 mm apart from *z* = 60 to *z* = −20. In darker blue areas, activation to deception identification exceeds activation to direction identification; and in red areas, activation to direction identification exceeds activation to deception identification (at *p* < 0.001 uncorrected). Key: a: premotor, BA6; b: parietal, BA40; c: medial frontal, BA6; d: anterior cingulate, BA32; e: posterior cingulate, BA23; f: dorsolateral prefrontal, BA46; g: caudate nucleus; h: superior temporal gyrus, BA37; i: anterior insula/frontal operculum, BA13/45; j: cerebellum; k: superior parietal lobule, BA7.

**Figure 5:**
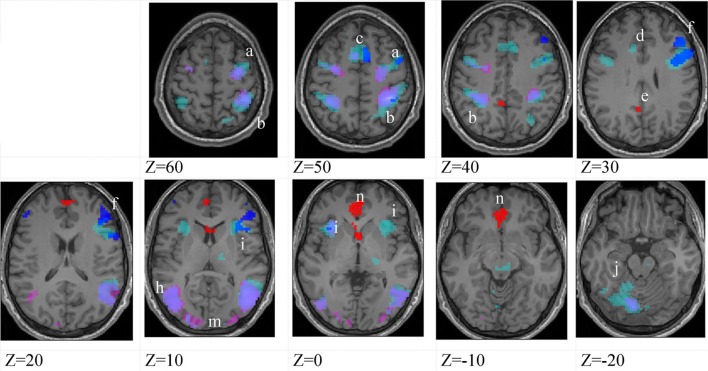
**Lower-skilled females**. Second-level fMRI activations (*p* < 0.005, FWE corrected, 25 voxels minimum cluster size) to deception identification (cyan) and direction identification (magenta) in point-light soccer video clips, relative to stimulus-matched non-biological motion (NBM) controls. Overlapping areas responding to both identification tasks appear purple. Activations above threshold (blobs) are displayed in co-registration with an individual normalized structural brain image and sampled in horizontal sections 10 mm apart from *z* = 60 to *z* = −20. In darker blue areas, activation to deception identification exceeds activation to direction identification; and in red areas, activation to direction identification exceeds activation to deception identification (at *p* < 0.001 uncorrected). Key: a: premotor, BA6; b: parietal, BA40; c: medial frontal, BA6; d: anterior cingulate, BA32; e: posterior cingulate, BA23; f: dorsolateral prefrontal, BA46; g: caudate nucleus; h: superior temporal gyrus, BA37; i: anterior insula/frontal operculum, BA13/45; j: cerebellum; k: superior parietal lobule, BA7; m: medial occipital cortex, BA18; n: anterior cingulate.

Figures 3–5 show group data for the activations due to the first-level contrast between the two soccer identification conditions and the NBM control trials, superimposed on horizontal sections of a normalized brain anatomy.

The data were entered into separate 2 × 2 ANOVAs, using the factorial design options of SPM8, One ANOVA for each participant group. Figures 3–5 show the responses in the deception task colored cyan (light blue), and the responses in the direction task colored magenta (pink). Overlapping activations are shown in a mixed color (purple). Both sets of data are based on first-level *t*-contrasts measured relative to NBM control. The second level data are displayed with a very conservative statistical threshold (*p* < 0.0005, FWE corrected) for both occlusion levels combined. Figure 2 shows results for higher-skilled males, Figure 3 for lower-skilled males, and Figure 4 for lower-skilled females. For all participant groups, the main anatomical areas showing strong activations were similar for deception identification and direction identification and included regions identified as part of a human AON specifically the intraparietal sulcus (BA40) and premotor cortex (BA6). The supplementary motor area in medial frontal cortex (BA6) was also consistently activated, along with the adjoining anterior cingulate cortex (ACC; BA32). Consistent activations in the anterior insula (BA13) were also present. The numerical data corresponding to Figures 3–5 are available in Tables 3–5.

**Table 3:** **Locations of significant clusters as shown in Figure 3**.

	**Higher-skilled males**											
	**Deception**						**Direction**					
	***BA***	***x***	***y***	***z***	**Peak *t***	**Cluster**	***BA***	***x***	***y***	***z***	**Peak *t***	**Cluster**
L insula	13	−33	17	4	13.1		13	−30	17	4	9.8	108
R insula	13	33	20	−2	15.1		13	30	23	−2	11.3	96
medial frontal	6,8	3	17	49	11.6	306	6,8	3	17	49	8.2	
anterior cingulate	32	6	23	34	10.7		32	12	17	40	8.3	
	32	9	17	40	9.9							
L premotor	6	−24	−7	55	16.6	856	6	−21	−7	55	19.4	557
	6	−39	−1	37	11.0							
R premotor	6	30	−7	55	15.2	1760	6	30	−7	55	15.4	1646
	6	42	5	40	12.8							
L caudate		−15	−7	19	7.7			−15	8	7	7.3	
L thalamus	VAN	−12	−1	4	8.7	100	VAN	−12	−1	4	7.7	
	VLN	−12	−19	16	8.0							
R thalamus							LPN	15	−22	16	8.3	40
L parietal	40	−39	−46	58	13.0	957	40	−33	−46	55	11.6	688
	40	−30	−49	55			40	−21	−49	55	11.4	
	40	−39	−43	43			40	−24	−58	55	10.4	
R parietal	40	36	−43	49	15.6	1404	40	33	−43	49	13.5	
	40	45	−37	58	12.8		40	39	−34	55	12.3	
	40	36	−52	43	10.4							
L fusiform	20	−42	−58	−23	11.3							
R fusiform	20	45	−52	20	8.7							
R mid temporal	21	54	−49	7	9.9							
L temporal	19,39	−48	−70	13	16.1	355	19,39	−48	−70	13	18.6	321
	39	−48	−58	10	11.6		39	−45	−61	10	12.1	
R temporal	37,39	48	−61	7	13.5	589	37	51	−64	4	13.9	459
							39	45	−58	10	12.0	
L cerebellum	6 L	−33	−58	−29	12.2		6 L	−27	−55	−29	11.2	242
	6 L	−12	−73	−26	13.95	589	6 L	−9	−70	−29	11.1	
R cerebellum							6 R	6	−73	−26	8.0	
R occipital	18	30	−91	7	7.9	39	18	30	−91	7	9.7	62
	18	24	−91	−2	7.8		18	24	91	−2	9.1	

*VAN, ventral anterior nucleus; VLN, ventral lateral nucleus; LPN, lateral posterior nucleus*.

**Table 4:** **Locations of significant clusters as shown in Figure 4**.

	**Lower-skilled males**											
	**Deception**						**Direction**					
	***BA***	***x***	***y***	***z***	**Peak *t***	**Cluster**	***BA***	***x***	***y***	***z***	**Peak *t***	**Cluster**
L insula	13	−33	17	−2	10.8	118	13	−33	17	−2	10.1	78
R insula	13	30	23	4	12.6	806						
Medial frontal	6	0	14	49	12.9	187	6	0	14	49	11.4	95
Anterior cingulate	32	6	23	31	6.7							
L premotor	6	−24	−4	58	11.2	99	6	−24	−4	58	17.2	296
	6	−36	−4	55	9.3		6	−33	−4	55	11.4	
R premotor	6	33	−4	55	11.0		6	33	−4	55	14.0	756
	9	45	5	31	12.5		6	24	−7	67	13.9	
							6,9	48	8	34	12.0	
L parietal	40	−33	−52	58	11.6	434	40	−33	−52	58	14.3	757
	40	−54	−40	37	11.1		40	−30	−43	46	12.7	
	40	−33	−43	46	9.9							
R parietal	40	39	−49	40	12.4		40	36	−49	52	12.3	655
	40	30	−49	40	11.0	469		39	−37	43	12.0	
	40	63	−34	31	7.7	27						
R temporal							37,21	54	−58	4	14.5	359
L temporal	39	−27	−58	−29	8.5	38						
	39	−42	−61	−29	7.0							
R temporal	37	54	−58	4	12.5		39	45	−67	7	13.0	231
	37	48	−67	−5	11.6		37	45	−64	−5	12.1	
							39	39	−67	13	7.2	
L sup parietal	7	−12	−73	49	8.0	32	7	−24	−52	64	11.1	
	7	−9	−67	58								
R sup parietal	7	12	−73	55	10.9		7	12	−73	−23	10.7	
Post. cingulate	23	−6	−55	−20	6.9							
Medial occipital	18	−9	−76	−23	8.6	66						
	31	−18	−58	−23	6.7							
Cerebellum							6 L	−9	−73	−23	9.4	166
							6 L	−27	−55	−29	7.4	
							6 L	−6	−55	−23	7.3	

**Table 5:** **Locations of significant clusters as shown in Figure 5**.

	**Lower-skilled females**											
	**Deception**						**Direction**					
	***BA***	***x***	***y***	***z***	**Peak *t***	**Cluster**	***BA***	***x***	***y***	***z***	**Peak *t***	**Cluster**
L insula	13	−20	17	4	14.5	167	13	−33	20	1	8.9	53
R insula	13	33	20	7	12.3		13					
Medial frontal	6	−6	8	52	14.5	287						
	6	0	20	46	10.9							
Anterior cingulate	32	−9	20	34	8.3		32	−6	8	52	9.6	32
L premotor	6	−42	−1	46	12.0	252	6	−30	−7	52	13.2	153
	6	−30	−7	52	11.7		6	−42	−1	46	8.5	
R premotor	6	27	−10	55	14.1	1041	6	27	−13	55	13.9	191
	6	45	5	31	12.9							
R thalamus	VLN	15	−16	7	6.7	43						
R thalamus	VPLN	24	−25	4	7.7							
L midbrain	S nigra	−3	−28	−8	7.6							
R midbrain	S nigra	9	−25	−14	7.7	48						
L parietal	40	−36	−43	46	14.1	377	40	−36	−43	46	10.6	207
R parietal	40	36	−40	49	23.6	673	40	36	−40	49	20.9	398
L occipital	19	−42	−67	13	11.9	293	19	−42	−67	13	13.0	470
	19	−24	−91	7	8.3		19	−27	−91	10	8.5	
R temporal	39	45	−58	10	16.5	641	39	51	−70	7	15.7	576
	39	48	−70	7	15.8		39,22	45	−58	13	15.1	
L cerebellum	6 L	−9	−76	−20	13.0	555	6 L	−12	−73	−17	9.8	83
	6 L	−30	−55	−29	11.5							
R cerebellum	6 R	36	−58	−26	9.0	36						

*VLN, ventral lateral nucleus; VPLN, ventral posterior lateral nucleus*.

Areas showing stronger activation to deception than direction identification are shown in darker blue; and areas showing stronger activation to direction than deception identification are shown in red. These are displayed with a liberal statistical criterion (*p* < 0.001 uncorrected, minimum cluster size = 5); some of these clusters coincide with the principal task-sensitive areas but some do not. A further analysis of task differences will be given in the next section of the Results.

#### Analysis of differences between identification tasks and expertise groups

To establish the significance of differences in between expertise groups and tasks, fMRI data were combined in a factorial ANOVA. There were three expertise groups (higher-skilled males, lower-skilled males, and lower-skilled females), two levels of task (deception identification, direction identification), and two levels of occlusion (0, −160 ms). The inputs to the second level factorial model were the first-level *t*-contrasts between the identification conditions and the NBM control condition. There were significant main effects of expertise group, task, and occlusion. No significant two- or three-way interactions were found.

**Figure 6:**
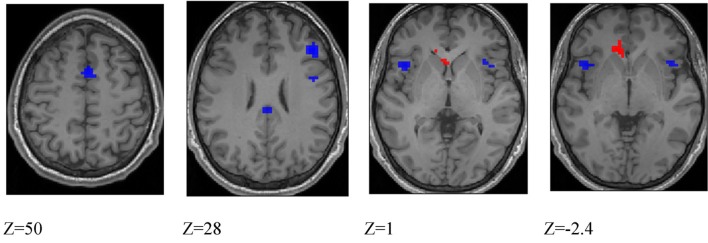
**Blue colored voxels indicate regions where activation is significantly greater for deception identification than for direction identification, and red voxels indicate regions that respond more to direction identification than deception identification**. *z* = 50; medial frontal, *z* = 28; dorsolateral prefrontal cortex and posterior cingulate cortex, *z* = 1; blue: left and right anterior insula, red: left caudate nucleus. *z* = −2.4; blue: left and right anterior insula; red: anterior cingulate cortex. Data are combined across participant groups and occlusion levels.

***Differences between deception and direction identification***. Figure 6 shows areas responding differentially to the two tasks, measured across all participants. Regions responding significantly more strongly to deception than to direction identification were identified in second-level SPM *t*-contrasts, at *p* < 0.05 with whole-brain FWE correction and minimum cluster size of 5. As identified in Table 6, these comprised the right dorsolateral prefrontal cortex (BA46), medial frontal cortex (BA6), right premotor cortex (BA6), left and right anterior insula (BA13), posterior cingulate cortex (BA23), and right intraparietal sulcus (BA40). Regions responding more to direction than to deception identification were limited to the (ACC: BA32) and caudate nucleus: the peaks in these two structures were connected at the cluster level at *p* < 0.05 FWE.

**Table 6:** **Peak activations at ***p*** < 0.05 FWE corrected**.

	***BA***	**Cluster**	**Coordinates**	**Peak *t***
**DECEPTION > DIRECTION**				
Medial frontal	6	36	6, 11, 49	6.3
R dorsolateral prefrontal	46	62	45, 29, 25	5.8
L insula	13	21	−42, 14, 1	5.6
R insula	13	53	33, 20, 10	5.3
	13		45, 14, −5	4.9
	13		36, 17, 1	4.7
R premotor	6	42	42, −1, 31	5.3
Posterior cingulate	24	8	−3, −31, 28	5.0
R parietal	40	5	60, −37, 31	4.9
**DIRECTION > DECEPTION**				
Anterior cingulate	32	53	−12, 20, −2	5.9
Caudate nucleus		53	−3, 20, 1	5.0
**LATE > EARLY OCCLUSION**				
L premotor	6	151	−33, −22, 70	6.0

Additionally, there was a significant main effect of occlusion that was represented by a single large cluster located in left premotor cortex (BA6).

**Figure 7:**
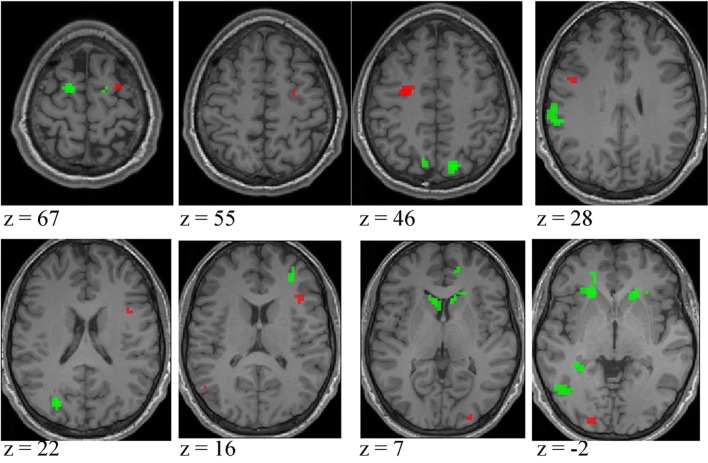
**Activation differences between higher- and lower-skilled males (red) and between lower-skilled males and females (green) in soccer action identification tasks at *p* < 0.05 FWE corrected, minimum cluster size = 5 voxels**. Data are combined across task types (deception identification and direction identification) and occlusion levels (0 ms, −160 ms). Second-level group analysis is based on first-level contrasts between identification tasks and NBM controls.

***Expertise group differences***. As shown in Table 7 and Figure 7, significant differences between higher- and lower-skilled male groups were restricted to task-sensitive AON regions: dorsal and ventral premotor cortex and frontal operculum, together with the left occipital-temporal junction, a region sensitive to visual motion, and some differences in occipital cortex. There were no significant voxels for greater activation in low- than in higher-skilled male players. Differences between male and female low skill participants were more extensive, principally comprising AON regions, but not overlapping with the male skill-related activations. The reversed *t*-contrast found areas in the temporal-parietal junction (BA19) and visual cortex (BA18) responding more strongly in female than male lower-skilled participants.

**Table 7:** **Expertise group differences at ***p*** < 0.05 FWE corrected**.

	***BA***	**Coordinates**	**Cluster**	**Peak *t***	**Coordinates**	**Cluster**	**Peak *t***
		**Higher-skilled males > Lower-skilled males**			**Lower-skilled males > Lower-skilled females**		
L premotor	6	−27, −7, 46	40	7.3	−15, −1, 73	48	6.0
	9	−42, 2, 28	10	5.0	−21, −4, 61	48	5.7
R premotor	6	30, −1, 67	6	5.3			
	6	27, −10, 55	5	4.8	18, −7, 61	16	5.5
R prefrontal	10				33, 41, 13	21	5.8
R frontal operculum	44	39, 8, 22	5	5.0			
R frontal operculum	45	39, 20, 16	10	5.0			
L anterior cingulate	32				−21, 44, −2	80	5.1
R anterior cingulate	32				15, 47, 4	36	5.2
L Parietal	40				−60, −34, 31	37	5.3
L superior parietal lobule	7				−12, −73, 43	54	5.3
R superior parietal lobule	7				15, −76, 46	14	6.1
L ventrolateral temporal	21				66, −19, −14	17	5.1
L temporal occipital	19	−54, −67, 13	13	5.8	−27, −76, 22	27	6.1
	37				−45, −67, −5	43	5.8
L occipital	18	−24, −94, 1	17	5.5			
R occipital	18	27, −94, 7	6	5.0			
L caudate nucleus					−21, 26, −2	80)	5.8
R caudate nucleus					18, 26, −2	36	5.6
Cerebellum anterior lobe					−21, −58, −29	6	5.0
					**Lower-skilled females > Lower-skilled males**		
L temporal	39				36, −64, 25	23	5.4
R temporal	39				45, −65, 16	70	6.2
L occipital	19				−15, −97, 19	31	5.8
	18				−21, −91, −2	12	5.2
R occipital	18				15, −85, 16	18	5.8
	18				24, −94, 7	5	5.1

## Discussion

### Responses to soccer action identification

The general pattern of activations found in the action identification tasks in the present study is consistent with previous research on action identification in general (Decety and Grèzes, 1999; Rizzolatti and Craighero, 2004; Filimon et al., 2007) and in fMRI sport anticipation studies in particular (Wright and Jackson, 2007; Wright et al., 2010, 2011).

The involvement of limbic and subcortical structures in soccer action identification, specifically, anterior insula, ACC, cerebellum, posterior cingulate cortex, caudate nucleus and thalamus, extends previous findings and suggests that there is an affective aspect to these tasks that is emphasized by the inclusion of deceptive stimuli (Grèzes et al., 2004; Molenberghs et al., 2012; Bishop et al., 2013). There is a clear correspondence in present results with an extension of the action observation (AON) brain network that has been identified as the “social network” (SN) (Grafton, 2009; Juan et al., 2013). It is evident from the within-groups analysis of fMRI data, employing control stimuli closely matched in all respects, that the SN network is not simply an accessory to AON but was strongly activated in both deception identification and direction identification in the presence of deceptive stimuli (Figures 3–5 and Tables 3–5). One of the largest and most consistently activated clusters in both groups of male participants and both tasks was in the anterior insula, and this area was also implicated in the females' data.

### Expertise and gender differences

Expertise differences between the male lower- and higher-skilled groups were reflected in substantial differences in accuracy across all tasks and conditions. In the fMRI experiments, expertise effects in the male groups were identified in a subset of the AON regions that were activated by the experimental tasks, consistent with previous research (Wright et al., 2010, 2011).

The male and female lower-skilled groups did not differ significantly in accuracy on behavioral measures, but comparison of male and female lower-skilled groups revealed significant differences in fMRI activation in both AON and SN structures. The female participants' lower familiarity with soccer actions and the very low level of soccer playing experience (Table 1) clearly differentiates them from the lower-skilled male group. This would arguably contribute to the observed expertise-related group differences in fMRI when comparing the two lower-skilled groups. An exception was found in visual cortex, including presumptive visual motion areas, where stronger activation was found in females than in lower-skilled males. This however, is consistent with Wright et al. (2011) where, in a badminton direction identification task, stronger activation in novice brains was found, exceptionally, in visual cortex.

An interesting question is whether the gender-specificity of the video material may be a factor. For females, the gender of viewer and performer was always different, and the argument would be that this may reduce AON activation. Calvo-Merino et al. (2006) recorded fMRI while male and female dancers viewed videos of both gender-specific and gender-nonspecific ballet moves. The strength of activations depended both on motor expertise and on the gender of the viewer relative to the gender of the performer. Separating these effects; they showed that motor experience of gender-specific movers increased activation in motor components of AON (premotor cortex, parietal cortex, and cerebellum). The increased activation of visual cortex in females relative to lower-skilled males does not contradict Calvo-Merino et al. (2006) whose results applied specifically to motor-related rather than visual areas of AON. In the present study, the effect of viewing a performer of the same or different gender is likely to have been reduced but not abolished by the point-light representation (Pollick et al., 2005; Calvo-Merino et al., 2010). The motor expertise effect is moreover a plausible one for the interpretation of the present results because the female group had substantially less motor experience of soccer moves than the lower-skilled male group.

Together with previous work, our results suggest that increasing familiarity with observed actions as well as motor experience of those actions is associated with increasing expertise and results in a shift in brain activation away from visual brain areas and toward AON motor areas and SN areas.

There are some general limitations of current fMRI research into action observation in general and sporting expertise in particular (Mann et al., 2013). The whole-body sensory-motor coupling, affordance-rich environment, and powerful contextual cues in soccer field play greatly exceed what is available to an immobile viewer of videos in a scanner. It is a challenge for future research to study the neural basis of sporting expertise in more dynamic and interactive scenarios. Despite this limitation, research to date has shown a consistent relationship between anticipatory behavioral responses to sports video and expertise in open-skill sports, and this extends to the use of point-light video stimuli (Abernethy et al., 2001, 2008; Huys et al., 2008). Moreover the present behavioral results recorded in the scanner (section Signal Detection Theory Analysis) have revealed expertise effects both in sensitivity (d′) and in response strategy (β). We would therefore, argue that the present fMRI results reflect the brain's processing of the minimum visual information sufficient to support an anticipatory response, and that our methods provide sufficient sensitivity to detect and localize expertise effects in the brain.

### Task-related differences in fMRI

There were no significant interactions between the task (direction identification or deception identification) and the three participant groups, either in the behavioral or in the fMRI data, but there were significant task-related differences in fMRI activations overall. Although AON and SN were activated strongly in both deception and direction identification, there were also significant differences in the two conditions (sections Within-Groups Analysis and Differences Between Deception and Direction Identification). The SN network is engaged particularly when participants are required to make inferences about the intentions of other people's behavior (Juan et al., 2013). This was explicitly the case in the deception identification condition, and in comparison with the direction identification condition, where participants were not required to identify deception, there was significantly greater activation in left and right insula and posterior cingulate, which are part of SN. It must also be fully recognized that despite the simplified and abstract nature of the point-light stimuli, they were universally understood as meaningful in a specific social context (the game of soccer).

The direct comparison of deception identification with direction identification in the present results provides further insights into specialization within the AON/SN network. First of all, there was significantly greater activation of ACC and caudate nucleus in the direction identification task compared with the deception identification task. The role of anterior cingulate has been established in response conflict and suppression of incorrect response tendencies (Carter and van Veen, 2007); and the caudate has been implicated in the learning of associations between stimuli and response tendencies (Melcher et al., 2013). This interpretation is consistent with both previous and present results; for example, Bishop et al. (2013) found ACC activity in higher-skilled players in the presence of deception, at a very early occlusion level, −160 ms. Bishop et al. (2013) also proposed that enhanced caudate activation in experts when predicting direction at very early occlusion (−160 ms), prior to an oncoming opponent's change of direction, indicated the learning of response contingencies. In the present study, awareness that an automatic left or right response tendency may need to be corrected, according to whether the move appears normal or deceptive, would occur only in the direction identification task. Thus, the greater caudate activation when predicting direction, may arise because the close mapping of leftward and rightward movements to left or right sided responses, respectively, is contingent upon whether the move is deceptive or normal; conversely, the identification of a move as deceptive or normal was not contingent on movement direction.

There was relatively greater activation in deception identification in right dorsolateral prefrontal cortex, which has a strong relationship with top-down cognitive and attentional control (Fassbender et al., 2006). This would be consistent with an interpretation that deception identification requires cognitive effort, whereas direction prediction is a more automatic perceptual-motor task (Kibele, 2006). The fMRI results are also consistent with Ivanoff et al. (2008) who found increased activation in pre-SMA (medial frontal cortex, BA6) associated with criterion (β) effects in a motion coherence task.

The behavioral data identified the strong influence of the trial type (deceptive vs. normal) on accuracy, and found significant interactions with task type. It may be possible in future to conduct a finer-grained analysis of fMRI responses to normal and deceptive moves using single-trial blocks (Bishop et al., 2013) and multi-voxel pattern analysis (Norman et al., 2006) in order to study how normal and deceptive moves are classified, and how this classification interacts with other variables such as temporal occlusion and task type.

It is likely that for both higher- and lower-skilled players, deception identification is a less practiced skill, requiring greater cognitive effort, and that conversely, the ability to react to the trajectory of someone's body actions is to some extent based on general as well as sport-specific experience, and therefore, likely to have become somewhat automatic. Thus, for late-occluded sequences, the direction of a normal move was determined at significantly higher accuracy than identification of this move as normal, suggesting that if the valid direction cues can be picked up, they readily prime the appropriate response. However, analysis of the sensitivity (d′) which takes into account the proportion of incorrect responses to deceptive moves, showed similar but slightly lower overall sensitivity for direction identification compared with deception identification, which would be consistent with the similar global strength of fMRI activations seen across tasks.

Likewise, early-occluded deceptive moves gave rise to significantly worse than chance direction identification because lower-skilled players especially were not simply responding randomly but were misdirected by the deceptive cues. This was borne out by analysis of likelihood ratio (β). In the direction identification task, observers adopted a liberal criterion, that is, one which increases both hits and false positives, and this was interpreted as a direct response to directional cues—veridical cues in the case of normal moves and false cues in the case of deceptive moves. Conversely, in the deception detection task, male observers tended to adopt a conservative criterion on late-occluded stimuli, which means that they were biased toward judging such moves as deceptive, and this inflated their correct detections of deceptive moves and reduced their correct detections of normal moves. Their overall accuracy remained higher than that of the two lower-skilled groups, as revealed by the d′ measure. The ability to identify a move as deceptive however, does not guarantee that its true direction can be identified. Experts are known in some circumstances to delay their responses (Brault et al., 2012; Mori and Shimada, 2013), perhaps so that they can inhibit and correct their initial automatic reactions. This dissociation between performance on deception and identification tasks is consistent with the differing involvement of the components of the AON and SN observed in the brain imaging data.

### Conflict of interest statement

The authors declare that the research was conducted in the absence of any commercial or financial relationships that could be construed as a potential conflict of interest.
